# Legally Affective: Mapping the Emotional Grammar of LGBT Rights in Law School

**DOI:** 10.1007/s10691-022-09504-7

**Published:** 2023-01-10

**Authors:** Senthorun Raj

**Affiliations:** Manchester Law School, Manchester Metropolitan University, Manchester, United Kingdom

**Keywords:** Emotion, Law, LGBT, Pedagogy, Rights

## Abstract

The teaching of critical race, feminist, and queer theory generally, and of LGBT rights specifically, has developed into a discrete, contested, and politicised area of teaching in English law schools and beyond. While there is some academic discussion on the personal and political significance of ‘promoting LGBT rights’ within law schools, less considered is how ‘LGBT rights’ are shaped by the emotions of legal academics and how these emotions circumscribe what we imagine LGBT rights can and/or should mean in law and critical legal study. To illustrate this dynamic, this paper uses critical race, feminist, and queer scholarship alongside work in the emerging field of Law and Emotion to articulate the ‘emotional grammar’ of teaching a Gender, Sexuality and Law unit at an English law school. Turning to autoethnography, I use my emotional experiences as a methodological tool to explore how emotions co-constitute the pedagogical, political, scholarly, and personal registers of LGBT rights as a descriptive, critical, and normative pursuit. In doing so, I use ‘emotional grammar’ as a novel way to map how emotions structure legal pedagogies invested in pursuing LGBT rights. Emotional grammar illustrates how emotions structure: (1) the articulation of LGBT rights as an object of study in law; (2) the pursuit of law to secure the rights of LGBT people; and (3) the shape of legal critique relating to LGBT rights.

## Introduction

Lesbian, gay, bisexual, and transgender (LGBT) rights^1^ has become an increasingly popular area of study in law schools. Once shunned as inappropriate or irrelevant to the study of law, topics relating to gender and sexuality are now popular, discrete units that students can select as part of an undergraduate law curriculum. For those without specific LGBT rights units/modules, ‘LGBT issues’ have also been incorporated into existing core LLB Law units, especially within law schools that aspire to engage students in a critical study of law.

In English law schools, so-called ‘Gender, Sexuality and Law’ (GSL) units largely reflect the incorporation and promotion of LGBT rights as an important socio-legal area of concern.^2^ This kind of unit is shaped by institutional aspirations for ‘research-led teaching’ (where GSL scholars can teach their research), ‘interdisciplinarity’ (where GSL scholars approach legal issues using intellectual resources from other disciplines), and ‘impact’ (where the research and teaching agenda of GSL scholars resonate with community, policy, media, etc.). Yet, GSL units also expose the vulnerability of both LGBT scholars and issues, as GSL teachers must navigate current university demands to rationalise content, expectations to expose their identities to classroom scrutiny (Robson 1998), and political accusations that these units are ‘promoting ideological agendas’ and ‘censoring unpopular views’ (Butler 2021). This article draws from that space of vulnerability – to think with feeling – in order to unpack the co-constituted personal, pedagogical, scholarly, and political registers of teaching LGBT rights. Currently, there is some research on the scholarly, political, and activist significance of promoting LGBT rights within law schools (Robson 1998; Ashford 2021; Ricciardo et al. 2021), ethnographies of teaching critical legal studies (Williams 1991; Boodia-Canoo 2020)^3^ and (auto)ethnographic literature on teaching with emotions in law school (Maughan 2011; Jones 2019; Raj 2021). What is less considered, however, is how emotions structure the teaching of LGBT rights in a law school and how this affective context circumscribes what LGBT rights comes to mean as a legal object as well as a mode of legal study. I seek to remedy this gap in the literature.

Undergraduate law units that focus on LGBT rights have emerged from students and teachers who have fought for content that reflects their personal lives, academic interests, community affiliations, and political engagements. The above pedagogical commitments generate optimism, anxiety, anger, frustration, and joy. It is timely then to ask how GSL teachers engage with these/their feelings and their consequences in knowledge production. This goes beyond merely questions of our emotional wellbeing. The design and delivery of GSL modules draw attention to different spaces of pedagogy where emotion matters: the initial selection of legal issues/topics to teach within a confined module, the kinds of technologies that are utilised to teach those topics (especially with the shift to online teaching as a result of COVID-19), the kinds of dialogues generated in response to those topics and technologies, and the way teachers navigate the evaluation and justification for their programmes after they are delivered. Moreover, these units and scholars who teach them cannot be neatly homed within the law school: many are stigmatised as ‘activist’ or ‘political’ for making LGBT rights a topic of formal legal study while academics who teach LGBT rights find themselves ambivalent about law’s role in addressing LGBT rights or feel complicit with legal (education) institutions that marginalise LGBT people.

The vulnerability and ambivalence are generative, and these emotional dynamics warrant elaboration. From this site of emotional contestation, this paper offers an elaboration through the concept of ‘emotional grammar’ of LGBT rights teaching. The first section selectively sketches the field of feminist and queer theorising in GSL scholarship to show how emotions become apparent within the field. The second section then outlines the turn to emotion in legal scholarship and pedagogy. The third section examines how these interrelated fields of scholarship relate to autoethnographic methodologies, with a particular focus on critical race, feminist, and queer theory in cultural studies scholarship. This paper engages in a “politics of feminist citation” by thinking with critical race, queer and feminist theorisations and methodologies of emotion to consider the place of emotion in LGBT rights teaching in law school (Ahmed 2017, 14–17).^4^ The conceptualisation of emotional grammar advanced in this paper emerges from threading Robyn Wiegman’s (2012) theorisation of ‘object lessons’ derived from identity-based knowledges with Patricia Williams’ (1991) articulation of ‘rhetorical events’ that occur when teaching critical legal studies, and Sara Ahmed’s (2006) ‘queer phenomenology’ of emotion and orientation. Each of these scholars, and their theoretical contributions, are shaped by racialised, gendered, and sexualised genealogies of intellectual and emotional labour.^5^ In bringing these distinctive contributions into conversation, this paper will show how LGBT rights teaching brings together the bodies of scholars (Wiegman, Ahmed, Williams, myself) and bodies of critical scholarship (queer, feminist, critical race) alongside emotions and legal rights. In doing so, I will show how mapping the emotional grammar offers GSL teachers a framework to navigate the personal, pedagogic, scholarly, and political registers that underscore the affective context through which ‘LGBT rights’ gets articulated (and contested) as a legal object, pursuit, and subject of study.

The final section provides an autoethnographic account of designing and teaching a GSL unit at an English law school to illustrate the emotional grammar of teaching LGBT rights.^6^ This approach uses emotions methodologically to analyse the relationship between personal, pedagogic, scholarly, and political registers in the production of LGBT rights knowledge. This paper embraces autoethnography to challenge the proposition that “useful knowledge” is only found by following canonical paths of doctrinal legal education or disembodied legal propositions (Ahmed 2019, 19). It concludes with a provocation to think through how we, as GSL scholars and teachers, ought to account for the emotional grammar of teaching LGBT rights. While this paper touches on the need to think about the place of emotion more broadly across the legal curriculum and law, I focus more narrowly in this paper on the issue of teasing out the specific relationship between our emotions and LGBT rights legal pedagogy. This is important so that GSL scholars might better understand our contested relationships to the law school, how emotions in legal pedagogies constitute LGBT rights in law as both normative and critical projects, and how those emotions render what we as GSL scholars imagine as possible and desirable in legal pursuits of LGBT rights.^7^

## Contesting Gender and Sexuality in Law

Before turning to emotions that underpin LGBT rights teaching, it is necessary to contextualise how gender and sexuality – including LGBT rights – are emotionally contested ideas within legal academia. Prior to the rise of Critical Legal Studies as a widespread disciplinary movement in the 1980s, legal curricula in Anglophone jurisdictions shunned discussions of gendered and sexual subjectivity, individual emotion, and social positionality as such considerations were considered to politicise the curriculum and threaten the objectivity and neutrality that were central to legal method (Unger 2015, 83–93). While this belief was not universally held, notions of objectivity, rationality, and dispassion framed dominant views not only about the exercise of law but also how law should be studied (Bandes 2021, 2429). In the social sciences, sexuality scholarship was ‘dirty work' because it related to abject topics (gay sex, bodily fluids, etc.) and scholars who engaged in it were stigmatised by their association to sexual perversities, including LGBT people or issues (Irvine 2014).

Over the last 30 years, however, legal academic denunciation of gendered subjectivity and sexuality has faded. Spurred by Critical Legal Studies alongside developments in Feminist and Queer Theory, numerous monographs, edited collections, and conferences have sought to highlight the existence (and production) of LGBT people within law while problematising their relationship with legal systems in the UK (Stychin 1995; Herman and Stychin 1995, 2000; Moran 1996, 2009; Raj 2020; Raj and Dunne 2020; Ashford 2021). Several law schools in the UK now offer specific units, including postgraduate degrees, specialising in gender and sexuality.^8^ English GSL scholarship has canvassed topics relating to the policing of homosexuality (Stychin 1995; Moran 1996), cruising (Ashford and Longstaff 2021), religious exemptions (Cooper 2019), asylum (Johnson 2011; Keenan 2015, 128–49; Bruce-Jones 2020), HIV criminalisation (Weait 2005), legal gender recognition (Sharpe 2002; Grabham 2010), prisons (Adams and Emmerich 2020), relationships (Maine 2022), and parenting (Harding 2015) to name a few. These scholarly engagements not only vary in theoretical orientation and methodological choices but also in their emotional disposition towards law. Some GSL scholars/hip involves doctrinal analyses of law with optimistic prescriptions for reform, such as scholarship that examines how law might be changed to allow same-sex couples to parent without discrimination or why trans people should be free to self-declare their gender without medical or bureaucratic hurdles. Other forms of GSL scholarship take a more pessimistic or critical posture to the legal system, such as scholarship that deconstructs how criminal law is structurally biased against ‘queer’ minorities or how categories of sex/gender are registered for the purpose of family law. These differences in emotional orientation, however, are not static or mutually exclusive. They reflect varying emotional attachments, such as feminist scholarly aspirations that seek to understand and challenge how gender is socially conceptualised, structured, and hierarchised (MacKinnon 1989; Conaghan 2013), and queer theoretical desires to expose how social norms organise and marginalise bodies, relationships, and identities (Zanghellini 2014; Raj 2018a). Unsurprisingly then, ‘women’ (gender) and ‘LGBT people’ (sexuality) are affective subjects of concern in feminist and queer research. To foreground emotion in this scholarship is to recognise that these forms of scholarship are generated from concerns, angers, or fears about particular social injustices, anxieties about the efficacy of institutional responses to address these injustices, and hopes for alternatives to harmful social, legal, and political arrangements.

In reflecting on the emergence of GSL as a distinctive subfield of scholarly research in England and the creation of the cross-institutional Arts and Humanities Research Council Research Centre for Law, Gender and Sexuality, Leslie Moran (2009) argues this “field” of scholarship could better be understood as a “home” (309).^9^ That is, as a recognised spatial arrangement in academia, GSL houses scholars who have shared commitments to not only expose patriarchal and heteronormative biases in law but engage in social justice work to challenge those biases (Moran 2009, 310). Despite some shared social and political commitments, the GSL home is characterised by emotional tensions that reflect different relationships to critical and normative legal projects. While some strands of feminist legal scholarship and LGBT equality theory seek to (re)imagine law as a vehicle of social transformation if only women and LGBT people were included as subjects of the legal system (Hunter 2010; Hunter et al. 2010; Chelvan 2013), queer and critical feminist scholarship queries the ability of the state to facilitate desired social transformation (Cooper 2019; Raj 2020; Renz 2020). These scholarly differences emphasise that GSL was not, and never can be, a romanticised home free of tension or debate.

In order to delineate a particular topic or subject in GSL, then, we need to pay careful attention to the objects of study, methodological choices, and modes of description and critique that constitute what Robyn Wiegman (2012) would describe as its “scholarly grammar” (30, 60). For example, the English Feminist Judgments Project seeks to “inaugurate a new form of critical legal scholarship, one which seeks to demonstrate in a sustained and disciplined way how judgments could have been written and cases could have been decided differently” (Hunter et al. 2010; Hunter 2010, 3). Here, the contributors to the project take appellate judgments as their object of study and deconstruct legal constructions of sex and gender within them to reimagine how they might have been decided differently, in a way that affirms the lives and experiences of the women in each of the cases. This critical reimagining, however, is not without constraint, as it is “disciplined” through a conventional form of judgment writing (limiting the judgment to the issues raised by the parties directly, consideration of precedents relevant at the time, etc.) that could be easily accepted by an English judge of the High Court, Court of Appeal or Supreme Court (Hunter et al. 2010, 3–29). Queer scholars have also drawn inspiration from the Feminist Judgments Project to rewrite judgments from a queer perspective (Sharpe 2017, 2018; Gonzalez-Salzberg 2019). Alternatively, the forthcoming Queer Judgments Project frees itself from the conventions of traditional judgment writing where contributors are invited to reimagine judgments relating to LGBT people and reimagine how those judgments might be expressed in a different form, which may be doctrinal, artistic, and/or theatrical (Queer Judgments Project n.d.).^10^ Accounting for the varied disciplinary grammars these interventions use requires paying closer attention to emotion.

## Cultivating Emotions in Law

The turn to emotion in legal scholarship has been shaped by feminist and queer scholars who question the systematic ways ‘feminine’ or ‘queer’ aspects of law like vulnerability, compassion, love, experience, subjectivity, and embodiment are sidelined or opposed by doctrinal or technocratic accounts of law that value abstraction, authority, objectivity, and neutrality (Bandes 1999; Nussbaum 2004; Maroney 2006, 2011; Raj 2020). Emotions are pervasive in law. They circulate across legal actors, institutions, spaces, disciplines, and professions. As Terry Maroney (2006) observes in her “taxonomy of an emerging field,” Law and Emotion analysis typically involves: (i) a focus on how an emotion can or should be reflected in law; (ii) identifying the legal mechanisms that express emotion; (iii) elaborating particular theories of emotion; (iv) examining how legal doctrine sediments emotion; (v) analysing the relationship between theories of emotion and law; and (vi) exploring how legal actors are or should be influenced by emotion (125–133). This nascent field of scholarship cuts across disciplines like sociology, psychology, neuroscience, behavioural economics, and philosophy and is characterised by disparate normative, methodological, and conceptual commitments (Shaw 2019). Emotion is an interdisciplinary concept: it refers to an unstructured bodily movement (Deleuze 1992, 626), a psychological process (Wiener et al. 2006, 234), and modes of impression (Ahmed 2004, 6).^11^ This critical scholarship on emotion is “epistemologically challenging” as it refuses to privilege objectivity or rationality (Abrams and Keren 2010, 1999) and does not subscribe to the myth of “judicial dispassion” (Maroney 2011, 636).

Emotions are also relevant to the teaching of law. Legal education research has examined emotion through student outcomes, module delivery, and assessment matrices. Caroline Maughan (2011) argues that affect, unlike cognition, is not part of a formalised system of legal study and notes some discussions of emotion in the legal classroom present it as a threat to the development of propositional knowledge and reasoning – intuition and feelings are elusive, disordered, and messy (19). However, she notes ‘messiness’ is a site of learning. Emotions, particularly pleasure and curiosity, are relevant to student agency and can be a heuristic to scaffold student understanding about legal rules or issues (Maughan 2011). Emotional management is also central to student wellbeing, maintaining student interest, and curating engaging discussions (Ferris and Huxley-Binns 2011; Jones 2019). Emotional literacy or being able to understand the “emotional fabric” of law is a vital part of developing lawyers who understand the needs of their clients (Del Mar 2011, 187). In the pedagogic encounter, being vulnerable to one’s emotions is generative as it encourages empathy towards others and understanding of why/how issues are important to us.^12^

Emotions are central to feminist and queer theorising about legal pedagogy, as objects of study and modes of learning. Feminist invocations of the personal as political render embodiment and experience (such as reproductive labour and sexual intercourse) as objects of study to understand how they give rise to political or legal claims (such as maternal healthcare and rape laws) (MacKinnon 1989; Colker 1994; Kapur 2005). These objects of study emerge through scholarly desires to make sense of, and remedy, gender inequalities and harms (such as the effects of the criminalisation of abortion and sex work) (Scott 1991). Queer engagements with sexuality have explored how desire is turned into an object of legal regulation (such as criminalisation of homosexuality) through political, historical, and national discourses (such as monogamy, nationalism, and reproduction) (Foucault 1978; Rubin 1984; Berlant 1997). Such objects of study emerge through scholarly desires to expose heteronormativity and make space for non-normative sexual communities (such as the decriminalisation of homosexuality or affirmation of non-monogamous relationships) (Love 2009; Muñoz 2009; Raj 2018a).

Law and Emotion scholarship has also drawn attention to how emotion features as an object of legal scrutiny and underpins the way in which legal scholars engage with law reform issues. Emotion is core to legal rules, for example where passion and fear are relevant to gendered criminal law rules on provocation and self-defence (Lee 2003) and shapes the desires of lawyers, scholars, and activists interested in reforms and rights. For example, anger shapes demands to redress harmful gender stereotypes or human rights violations associated with those stereotypes in law (Lorde 1984; Brown 1995; Nussbaum 2016). Emotion is relevant to any discussion of legal actors, processes, spaces, institutions, texts, norms, rules, and habits (Maroney 2006).

Some recent legal education literature has hinted at the emotions that animate the inclusion of LGBT rights in law school. Chris Ashford (2021) has observed how the inclusion of gender and sexuality in the legal curriculum are not simply about the inclusion of additional topics. Gender and sexuality units reshape the architecture of a traditional law school because feminist and queer legal scholars hope to engage students in broader questions about activism, policy, and law reform rather than simply insisting on doctrinal study. GSL academics subvert the ‘professionalism’ of law school etiquette. GSL units encourage students to recognise and share relevant anecdotes, feelings, desires, and experiences that disrupt assumptions of the status quo (Ashford 2021, 454–7). In a comprehensive survey about international human rights law teaching relating to sexual orientation and gender identity, Paula Gerber and Claerwen O’Hara (2019) argue that critical legal teaching has come to embrace the lives of those the law seeks to speak about by paying close attention to language, identities, interdisciplinarity, different spaces of law, diverse issues and actors, and social contextualisation. Ruthann Robson (2018) writes that “queering” legal pedagogical conventions that assume a dispassionate teacher and disengaged student in a classroom complicates how GSL teachers imagine “successful” classrooms and scholarship (277). Others have attempted to foreground how LGBT students engage with legal education (Ricciardo et al. 2021).

The scholarship discussed above provides some important normative and critical context for locating emotions in the teaching of LGBT rights in legal curricula. Yet, the socio-legal context in which LGBT rights teaching takes place warrants greater emotional exposition and self-reflexive critique to understand the relationship between how LGBT rights in law become an object of study and how our study/teaching of those rights shape their broader circulation. Returning to Maroney’s taxonomy of Law and Emotions scholarship, this paper develops a theory of emotion that foregrounds the iterative relationship between emotions, legal pedagogy, and law. This approach has descriptive (what emotions do in LGBT rights teaching and law), critical (how emotions structure LGBT rights teaching and law), and normative (why emotions matter to LGBT rights teaching and law) dimensions.

## Emotional Grammars and Autoethnographies of Law

When it comes to developing a critical emotional pedagogy about LGBT rights in law schools, two texts are useful to help illustrate thinking with/about our vulnerabilities and emotions as legal teachers by using an autoethnographic methodology: Patricia Williams’ *The Alchemy of Race and Rights: The Diary of a Law Professor* (1991) and Ruthann Robson’s *Sappho Goes to Law School* (1998). In *The Alchemy of Race and Rights*, Patricia Williams uses a series of personal vignettes to offer accounts of teaching race/ism and property law in an elite American law school. What is significant about Williams’ book is the use of experience and emotion does not serve a moralising or normative gesture, rather it is an analytic device that seeks to “fill” or “connect” gaps between “my psyche and the readers’, between lived experience and social perception, and between an encompassing historicity and a jurisprudence of generosity” (Williams 1991, 8). Williams’ approach is a foundational element of Critical Race Theory where her personal narrative is combined with doctrinal analysis to flesh out the material impacts of racism – alongside intersecting experiences of sexism and classism – across individual, interpersonal, and institutional contexts (Williams 1988; Mirza 1999, 114). In other words, she uses her emotions as a Black woman and law professor to understand ‘the law’ as a series of rhetorical events constituted by specific relations of race, gender, class, desire, etc., including those within the classroom (Williams 1991, 11). In this sense, law is performative. It does not simply accrue force and meaning from judges who make declarations or legislators who authorise statutory text but also from legal scholars who research, analyse, and teach law (White 1985; Brooks and Gewirtz 1996; Coombe 2000). This also emerges from students who study law and then go into practice: performativity of law taught in classrooms (as doctrines, modes of interpretation, etc.) reproduces itself in chambers and courtrooms.

In *Sappho Goes to Law School*, Ruthann Robson reflects on how desire structures lesbianism in law and the pedagogical style of lesbian legal scholarship. The novelty of this intervention is she refuses to sideline the anxieties that emerge when discussing sex in a classroom and the “messiness” of her anxieties as a lesbian scholar in a heteronormative law school. Instead, her account helps to make explicit how lesbian sexual desires complicate the study of conventional legal topics (like indecency laws), particularly when students feel comfortable enough to disclose their personal sexual experiences when contributing to discussions of jurisprudence (Robson 1998, 215–24). Williams’ and Robson’s texts are illustrative of how marginalised scholars (based on their race, gender, and sexuality) are positioned in relation to the law school and how we might take feelings as a serious analytic category in law. They expose the emotional toll of such work – including the vulnerability and bravery – necessary to force a conversation to challenge law’s purported rationality and objectivity.^13^ Each account connects desire, emotion, and law at the individual level (of being a marginalised person or ‘outsider’ in law academia) and the institutional level (of teaching ‘outsider’ critical legal scholarship).

I take these texts together as a departure point to begin an emotional exegesis of LGBT rights teaching in law schools. To do this, we need to acknowledge that the legal classroom is a space that produces, not simply disseminates, what law teachers mean by feminism, queer, LGBT rights, etc. These learning spaces are co-constitutive of normative and critical projects, where bodies of knowledge (LGBT rights and queer scholarship) are produced alongside bodies of students and teachers (who might be LGBT) (Waite 2018). In other words, we can locate queer/feminist/LGBT rights theory in everyday relationships of curriculum design and teaching practice, not just in the abstract concepts or ideas published in elite scholarly research outputs or jurisdictionally specific legal texts (Brim 2020, 17). These spaces are embodied and fluid.

In order to illustrate how these affective teaching spaces constitute personal, pedagogic, scholarly, and political registers of LGBT rights, I coin the concept of ‘emotional grammar.’ I use emotional grammar here to refer to the emotions that both underpin, and are produced by, linguistic referents and mechanisms (spoken words, written texts, concepts) that communicate ideas and make them understandable to others. This grammar is a means of framing, shaping, structuring modes of communication in law schools, objects (cases, topics, rights) and methods (curriculum design, legal interpretation, classroom discussion). If the 'scholarly grammar' I described above refers to the disciplinary logics that constitute a field, then I extend this concept using emotional grammar to capture what constitutes the personal, scholarly, pedagogic, and political dimensions of a field (such as LGBT rights teaching in GSL) and their co-constitutive relationship to each other. In selecting some specific examples of GSL work that emerged from English legal academia and reading them alongside two autoethnographic accounts of ‘outsider’ critical legal teaching relating to race, gender, and sexuality, I highlighted how scholarly grammars of LGBT rights scholarship are inseparable from the powerful personal and political desires that animate the inquiries of scholars in the field (Wiegman 2012, 4, 60). There is a normative force to legal inquiries that make room for emotions and experiences that have been marginalised, as “reclaiming that from which one has been disinherited is a good thing” (Williams 1988, 6). Relatedly, all forms of knowledge production and study – such as legal knowledge produced through law school training – involve forms of “sustained attention” (reading, thinking, comprehending, reflecting, feeling, etc.) that produce the very objects of knowledge they purport to be paying attention to (Meyerhoff 2019, 13–4). If we are to grasp the object of LGBT rights (as a normative pursuit or critical project) we must attend to the emotions that animate grammars of LGBT rights pedagogies in law schools. We can engage in what might be described as “wench tactics,” where we slow down and linger with the affective moment to critically reimagine law and legal education without abandoning our commitments to either (Fletcher et al. 2017, 18–20).^14^

I turn to autoethnography as an a/effective way to render this emotional grammar. There is no single or simple definition of autoethnography. In this paper, I take autoethnography to mean personal storytelling of my emotional encounters of teaching LGBT rights combined with a self-reflexive discourse analysis of those emotional encounters.^15^ This is a feminist, critical race, and queer methodology. It is a feminist method because it disrupts the mind/body dualism by taking the scholar’s body-in-motion as both the object of study (emotions generated from teaching LGBT rights) and the method through which that object is analysed (emotions as the means to understand what the teaching of LGBT rights entails) (McRobbie 1982, 51; Johnson et al. 2004, 33). This approach is indebted to Critical Race Theory because it relies on everyday anecdotes (of being an LGBT teacher and a teacher of LGBT rights) as a means of confronting doctrinal dilemmas (the utility of LGBT rights) (Mirza 1999, 115). This method of research is also queer because it challenges the normative objective distance between an academic (LGBT law teacher) and their research subject/object (LGBT rights) and resists being “disciplined” into a static analytic method (Holman and Adams 2010, 197–8). Moreover, by making my body into the ‘body of knowledge’ central to this paper, this approach to generating scholarship makes porous the boundaries between what we know about LGBT rights from what we do as LGBT scholars from who we are as LGBT people (Waite 2018, 222).

As a form of research and analysis, autoethnography affectively connects the personal with the political, as well as the scholarly with the pedagogic. Autoethnography allows me to situate the researcher (me) within their field of study (LGBT rights) and combine personal observations or reflections (LGBT rights teaching) with literature that frames those observations (LGBT rights scholarship). The account that follows allows me to recount my personal experiences of teaching LGBT rights in an English law school to highlight how emotions are embodied in the classroom while combining this with discourse analysis to make ‘sense’ of how emotions scaffold LGBT rights as both a critical legal object and normative pursuit. In gathering ‘data’ for this paper, I turned to my unit guide, recorded lectures, weekly outlines, Flipgrid videos, and the reflective notes I made at the end of each class. By making personal experiences part of my research methodology, this paper might be better described as “sensuous scholarship” or “autotheory” because it attempts to utilise my emotions as a frame to think through broader political questions, pedagogic interventions, and theoretical ideas (Bondi 2005, 231–46; Clare 2015).

Echoing the autoethnographic work of Williams and Robson discussed above, I use this approach to challenge a conventional academic (legal) grammar that presupposes an objective distance between the scholar and their subject and a dispassionate mode of writing that privileges rationality over feeling (Johns 2004, 478). In making this claim, I do not assume my experience is universal or the specific emotions at play are generalisable beyond my encounters. Rather, the autoethnography that follows seeks to enable other GSL scholars to map individual emotional grammars of their teaching and how these grammars institutionalise the personal, pedagogic, scholarly, and political registers of LGBT rights in law and legal education.

By thinking and writing with emotion, I illustrate how emotional grammars thread together the personal, scholarly, pedagogic, and political registers of LGBT rights. To do this, I use Sara Ahmed’s concept of “queer phenomenology” to note how objects of study (like LGBT rights) cohere through repeated spatial and bodily relations. An object is discernible through logics of distance (how close or far away it is from us) and difference (how familiar or confronting it appears to us). These objects then form attachments that anchor us to particular spaces (Ahmed 2006, 11–23). For example, if we think of precedent as a legal object, we can observe how it emerges through judicial interpretations of previous cases that determine how proximate previous decisions are to a current adjudication (distance) and how familiar those case are to the facts under consideration (difference). Precedent then becomes an object to anchor how we (judges, lawyers, and legal academics) understand what the law is.

To expand on how LGBT rights, as a legal concept, becomes an emotional object of teaching and learning, I also riff from Williams’ (1991) concept of law as a ‘rhetorical event’ and Ahmed’s (2006) notion of objects as a ‘queer phenomenology.' In thinking about my attachments to both Williams’ and Ahmed’s writing, it is important to acknowledge how their scholarship gives expression to their emotional labours as racialised women struggling to write about racialised, gendered, and sexualised inequalities in academic disciplines that alienate them and dismiss the reproduction of their scholarship (Mirza 1999, 118; Ahmed 2017, 150). Williams’ writing critiques critical legal scholarship that dismisses legal rights altogether while Ahmed’s writing cautions against the abstraction of critical theories from the emotional encounters that gave rise to them. Bringing together Ahmed’s and Williams’ scholarship on law and critique gives voice to my struggles as a racialised, queer academic who pursues LGBT rights while critiquing this pursuit in law school.

In the following section, I explain how LGBT rights is an emotional event in law schools, one which we can only comprehend by taking account of its emotional grammar: acknowledging our emotions as embodied states (Sedgwick 2003), forms of labour (Hochschild 1983), and cultural politics (Ahmed 2004). Emotional grammar is a way to illuminate converging bodies of critical race, feminist, and queer scholarship with scholarly bodies articulating converging emotions in academic institutions. This allows us (as LGBT rights scholars) to foreground personal desires for pursuing LGBT rights through law, scholarly dispositions towards critiquing legal rights, pedagogical attachments to cultivating critical thinking in our students, and political commitments to affirming minoritised lives (including ours and our students) in law school.

## Teaching LGBT Rights in Law School: An Autoethnography

In 2020, during the midst of the COVID-19 pandemic, I was asked to redesign a Gender, Sexuality and Law unit while working at Keele University. This was not a completely unconstrained task. I was provided with a unit skeleton that had been approved to meet institutional requirements. I was tasked with elaborating the content of the unit. This unit would introduce final year undergraduate law students to a study of LGBT rights. It would also be delivered online through a mixture of pre-recorded lectures and interactive seminars. The unit had a broad aim with a limited set of learning outcomes:

“This module aims to introduce you to the concepts of gender and sexuality and to enable you to develop a critical perspective on the relationship between gender, sexuality and the law.

- Critically analyse the role law plays in the regulation of gender and sexuality.
- Demonstrate a critical and evaluative understanding of sexual and gender dimensions of law/legal studies.
- Apply theoretical knowledge to a series of ‘concrete’ socio-legal issues.
- Develop independent research and writing skills through completion of a research essay.”

Thinking about developing this unit in response to the aims and outcomes above prompted a series of questions. How might I conceptualise gender and sexuality? How would I define law? Which aspects of their relationship would I explore and how would I do that? Which feminist and queer theories would be useful? Which socio-legal methods seem most pressing? Which sources of law could I use? Would I limit the focus to the UK or take a more transnational focus? These questions were personal. Each of these questions provoked a range of feelings that led to personal, scholarly, pedagogic, and political reflections: feelings that spoke to different aspects of being an effeminate brown gay man, a queer legal scholar, a critical law teacher, and an LGBT rights activist within the space of the legal classroom.

When I started to reflect on redesigning a LGBT rights unit, my thoughts begin to oscillate quickly between local law reform debates I had engaged in related to ‘conversion therapy,’ that had generated anger and frustration, and more joyous imaginings of recent international campaigns relating to the advancement of marriage equality in Australia and the decriminalisation of homosexuality in India (Raj 2018b, 2020). But when designing the unit, I also felt a sense of fear and worry as I began to reckon with how COVID-19 had intensified the challenges for LGBT people who were now unable to seek asylum, trans folks who endured further delays to access healthcare, and those experiencing domestic and family violence because of stay-at-home orders (Amnesty International 2020). Whether joy, anger, or fear, my emotions functioned as orienting devices. They drew me towards specific topic areas, and those topics “pressed” themselves into me as worthy of inclusion in my module (Ahmed 2004, 11). These emotions were not simply individual physiological sensations but functioned as a social hermeneutic, one capable of connecting disparate issues – as well as students – together. This thought was energising: I felt hopeful at the thought of occupying a (virtual) classroom where my students and I could share our political attachments to LGBT rights amidst the insecurity of living through a pandemic and learning from home. Yet, I was also wary at the start of the unit about how I might discuss contentious topics with students who lacked similar political commitments or who held hostile views towards certain LGBT groups or sexual practices. In the virtual classroom, I found some students expressing their frustrations at lack of legal action (to make seeking asylum for LGBT people easier) while others shared hopes regarding previous reforms (to recognise same-sex families) while some were anxious about law’s (over)reach into their personal lives. These emotions connected most of my students to the topic and they also connected us to each other, as we reflected on how/why LGBT rights were made meaningful.

I want to turn first to the personal register through which ideas about race, gender, and sexuality became emotionally salient in the classroom. As an Asian gay man who is routinely read by others as feminine (owing to my camp expressions as a brown queer) I have always experienced race, gender, and sexuality as intimately connected. To think about my sexual desire or orientation is to imagine the race and gender presentation of those (often white men) whom I find attractive and the intimacies I wish to cultivate with them. To wonder about my gender is to think about bodily comportment and interactions where I have been chastised by classmates for a ‘gay walk’ or ridiculed by strangers for speaking with a ‘squeaky gay voice.’ To reflect on being brown is to imagine how my sexual currency is shaped by whiteness that both fetishises my brown body for some and renders it undesirable for others.^16^ Thinking about placing myself into the narrative of the unit – even as an introductory way to get students to think about the racialised and gendered politics of being LGBT – made me hopeful and nervous. I was hopeful, to echo Williams and Robson, that sharing personal experience would not simply be solipsistic but would be a hermeneutic for students to understand sexuality and gender. Yet, I was nervous at the prospect of rendering myself vulnerable to my students (including experiencing ridicule relating to my disclosures or contempt towards my ‘squeaky’ way of talking and flamboyant gait when walking around the classroom). In a university context which maintains boundaries of professionalism that distance the student from teacher, I was unsettled by the prospect of too much disclosure (Nichols 2018, 43). I also felt uncomfortable about creating a space where students are asked to share sensitive personal information related to their protected characteristics (such as race, sex, gender identity, and sexual orientation), which may also trigger associated traumas. My anxieties here were borne through a speculative anticipation of my students’ anxieties. Here, I think of anxiety as an emotion that exposes the possibility of trauma or the repetition of a previous trauma (Salecl 2004, 120). Some LGBT students might not wish to be ‘out’ to others (even online) for fear of experiencing shame or hostility. Other LGBT students might find it wearing or difficult to speak about issues they experience directly. Being at an online distance to my students also made it harder to cultivate a pastoral intimacy. This might make students less willing to seek support.

In thinking with these anxieties, I did not ask students (or myself) to disclose anything specific about their sexuality or gender as a way of introducing themselves. Rather, I prompted students prior to the introductory seminar to share a text that could evoke discussion of these issues. Students were asked to record a 1-minute video in response to the following question: “What text illustrates something you find interesting about gender or sexuality?” Students responded generously by sharing stories of their favourite books, songs, memes, films, and news articles including Lady Gaga’s *Born this Way* (2011) pop anthem and the Ryan Murphy series, *Pose* (2018). Students spoke about gay sex, queer friendship, and gender non-conformity in candid and casual ways. As I watched each video, I experienced a sense of excitement when hearing about the richness of the texts my students had chosen as they described learning about gender and sexuality generally through experiences of minoritisation (with some of them including their own experiences of being gay or trans and coming from ethnic and religious minority communities). In doing so, they ended up answering what it meant to be LGBT. Most notably, a sense of pleasure emerged in response to ‘reading’ and hearing the excitement by which they described their text (both in the impassioned tone of their talking and vibrant hand gesticulations that punctuated their talk). By using popular culture to make sense of gender and sexuality, as both an idea and identity, I felt a greater sense of intimacy with my students as I was able to observe how gender and sexuality were “lived” through their negotiation of Anglophone cultural texts or objects (McRobbie 1982, 50). By taking the racialised, gendered, and sexualised self as the starting point for the class, I was affirming the (critical race feminist) methodological importance of grounding these ideas emotionally for my students through personal interest rather than arriving to them via legal problems or philosophical explanations. Making space for this pleasure in the legal classroom – to allow students to share what animates them and to take that seriously – was a queer intervention to more formalised aspects of legal study that prize neutrality, objectivity, and dispassion.

In terms of a personal register, my emotional exploration of what is meant by gender and sexuality was indexed by what it meant personally to be LGBT even if this was refracted through a narrow set of cultural texts that refract whiteness.^17^ To know what it means to be LGBT preceded how we talked about LGBT rights in the classroom. Here, communication and understanding of these ideas were made possible by the personal pleasures and anxieties associated with responding to popular culture and discussing them (and ourselves) through video recordings. To think of this as an ‘emotional event’, as outlined above in relation to Williams’ and Ahmed’s work, is to pay attention to how gender and sexuality get articulated here through our embodied states (feelings of pleasure at discussing American pop culture we enjoy watching or listening to), emotional labour (managing the expression of our pleasures when discussing sex and the associated anxieties that emerge in anticipation of disclosing too much personal information), and cultural politics (the expressed pleasures and anxieties circulating between bodies and cohering in the virtual classroom as they emerge through discussion of texts, and associated gesticulations, related to gender and sexuality).

The emotions described in personal terms above also connect to a scholarly and pedagogic register. As a legal researcher who specialises in LGBT rights, I felt relieved at the thought of being able to teach my research, instead of having to teach a core unit outside my research area that would require significant preparation. At a time when I (and many other colleagues) were scrambling to shift entire programmes online, I felt relief at having my teaching and research agendas align. As a critical law teacher, designing an elective rather than core LLB unit, I did not have to fit the unit into a specific professional framework (such as the Solicitor’s Qualifying Exam). I was not only personally unburdened but the unit itself was freed from institutional constraints of conceiving law solely in doctrinal terms or having to limit the scope of study to certain topics relevant to the English jurisdiction. By thinking of gender and sexuality through pop culture, I could encourage students to think of law in queer terms, beyond the conventional registers of statutory interpretation, case law analysis, and legal textbook commentaries. The pleasures described above when discussing what it meant to be LGBT provided an emotional anchor to frame the law as a form of culture – one which takes shape through less conventional objects like art, television, film, music, books, memes, TikTok videos, etc.(Coombe 2000).

In the virtual classroom (hosted on Microsoft Teams), I would begin each session by asking students to share how an aspect of popular culture related to the week’s topic (same-sex families, LGBT people who seek asylum, medicalisation of trans people). Moreover, by using broader cultural texts to frame the topic, I was then able to draw on feminist and queer theories that derive from consideration of such literary and artistic texts and then explore how such theories relate to law broadly conceived (Berlant 2000; Love 2009). This also provided students with an opportunity to share an emotional connection to the topic – through their text or anecdote of choice – without necessarily having to disclose their own experiences (though some felt comfortably able to do so behind the ‘distance’ of the screen). For example, in a session on legal regulation of LGBT kinship and family, students anchored their reflections on the topic by parsing what they understood love to mean. Rather than conceive of the concept of kinship in categorical terms (nuclear, same-sex, single-parent, extended, etc.), students were motivated by the affective content of the love they felt when seeing certain family dynamics represented in film as well as from their childhood memories (references were made to care, nourishment, and sentimentality). I think of love as an attachment that involves “bonding with others in relation to an ideal” (Ahmed 2004, 124). Here, that bond is expressed through the statements students made about texts and anecdotes that represented idealisations of family to them (“This show made me yearn to be in that family”). In thinking about making room for love in the classroom, expressed here as an attachment to an ideal text, the pedagogic value of this becomes clear: students were motivated by that attachment to discuss issues of family and kinship. This love also registers the scholarly intervention: to render law’s shifting relationship to regulating family and kinship as one of affective cultural change. This also has a political dimension, as taking love as the primary basis of articulating family in the legal classroom, circumscribes the types of family structures/types that we imagine ought to be legally recognised (Raj 2020, 116–39). Love framed how we affirmed case law and legislation that lovingly extended recognition to same-sex partners/families (like the US marriage equality case,^18^) while condemning jurisprudence that did not.

Mapping the emotional grammar of this classroom encounter reveals the dynamic and co-constitutive relationship between personal, scholarly, pedagogic, and political registers of LGBT rights knowledge in law. Students’ embodied experience of love (towards a text or memory that represents an ideal family) is given expression through how they speak about their bond to that text in class. This love takes scholarly form (by shaping law as an object that can be studied in terms of how it conceives of love in specific kinship arrangements like same-sex families) and pedagogic relevance (the love of specific shows like *Pose* (Murphy et al. 2018) and *Modern Family* (Lloyd and Levitan 2009) generates student motivation to engage with the topic). This also has a racialised dimension, as texts like *Pose*, that one student referred to in class, showed how Black and Latinx trans and queer people make family, and express love, through dance, dress, and banter (casually referred to as a “Ball House”).^19^ The communicative aspect of this love also threads to a political register as how students imagine and then talk about love, and script its possibilities, circumscribe what law ought to recognise as appropriate or worthy (Berlant 2000, 438). Yet, mapping the emotional grammar of love here is not to celebrate it. My own personal and pedagogic motivations in making room for love are about encouraging student engagement and scaffolding a heuristic for them to understand law’s relationship to kinship and family. Yet, I was anxious about the scholarly and political dimensions of enabling expressions of love indexed to romance, monogamy, reproduction, and sentimentality (as evident in *Obergefell*) as they risked foreclosing recognition to those who embraced friendship, transience, and polyamory (Berlant 1997; Cossman 2008). Alternatively, I was also wary of how critiquing these loving expressions of conventional forms of intimacy risked romanticising counter-normative, ‘queer’ forms of non-legally recognised kinship (such as Ball Houses) primarily for the curiosity and ‘exotic appeal’ they inspire for my students who were cis, straight, and/or white. Parsing the emotional grammar of the encounter reveals the convergences and tensions that might emerge between teachers and students in a classroom discussion of LGBT rights as an object of study while drawing attention to how these objects of study (legal recognition of family) take shape through emotions underpinning desires to pursue LGBT rights (broaden legal recognition of family) and the emotional logics through which critiques of those rights are expressed (critiques of legal recognition of family).

The above examples of my experiences speak mostly to the personal, scholarly, and pedagogic dimensions of teaching LGBT rights: anxieties and pleasures shared between teachers and students. I want to now draw attention to how emotions also structure what LGBT rights can mean politically and how we might pursue this through legal education. As an LGBT rights activist, I encountered pain and anger when designing and teaching the module because of the political context in which I was teaching. My hurt and indignation at stalled efforts to reform the Gender Recognition Act and prohibit ‘conversion therapy’ in the UK had preoccupied me and this brought to fore specific topics that would form concrete socio-legal issues to be discussed in class.^20^ In an embodied sense, my anger took shape as a desire to strike back against the hurt generated at political inaction that stalled attempts at law reform to enable self-declaration of gender and prohibit a form of insidious torture of queer and trans people. This anger was animating, energising me to push for change (Lorde 1984, 127). Thinking of anger as a relational expression of movement and attachment, anger oriented my attention to these specific issues as “pressing” topics while the desire to strike back against the injustices they represented led to their inclusion as topics for discussion in the unit (Ahmed 2004, 11).

Here, the emotional grammar underpinning the design of the unit exposes how the political and personal registers in which these topics came to matter to me then contoured the form of their pedagogic and scholarly articulation. Each topic was not presented neutrally for discussion or debate but rather contextualised through different forms of anger: the personal testimonies of those LGBT people who angrily yearned for reform and those from a broader public who recognised the necessity to protect LGBT people from violence, discrimination, and abuse and were angry about political failures to realise this necessity. By contextualising the topics through anger, I engaged in a pedagogic encounter where students could relate to anger normatively when thinking about the wellbeing of LGBT people while using anger to think critically about law and law reform. This pedagogic use of anger fed into a scholarly register that focused on the desirability of using law to categorise people in terms of diverse gender identities or to formally designate their sex (Renz 2020) and the possibility of eliminating insidious conversion practices that degrade LGBT people through blunt criminal or civil sanctions (Ashley 2022). By mapping the emotional grammar of teaching LGBT rights, we can observe how LGBT rights are affectively rendered normatively important in law and legal education, but we can also note how we (as lawyers, legal scholars, and activists) pursue LGBT rights as affectively circumscribed by the possibilities and failures of law. Mapping the emotional grammar enables us to understand this tension within law and how it is held together emotionally.

## Conclusion

LGBT rights is an emotionally vexed subject/object in law and legal education. LGBT rights themselves are vulnerable to political attack while the teaching of those rights relies on the vulnerabilities of staff and students. As I have shown, this emotional space has been generative of both GSL scholarship and Law and Emotion scholarship, as both have emerged from contested relations of vulnerability. While this has not happened significantly to me, scholars undertaking either/both areas of research are vulnerable to stigmatising accusations of their work being messy, subjective, and even perverse while they seek to expose the vulnerabilities of individuals and institutions to law’s reach or seek to draw attention to law’s vulnerability to individual emotions and racist, sexist, heteronormative biases. This is not to suggest that emotions are experienced universally by those who teach LGBT rights nor are emotions pre-determined by specific teaching encounters. As Ahmed observes, emotions are not simply pre-social states involving bodily reactions or physiological drives but are the descriptions we give to our socialised experiences of movement and attachment. In thinking about these communicative experiences of movement and attachment in law, alongside the vulnerabilities of scholarly bodies associated with doing LGBT rights, we can attend to our emotions as legal teachers while reading (and responding to) the emotional encounters we have with our students. In doing so, we can ask how emotions produce LGBT rights as a legal object of study and account for the affective generation of this object of study in order to understand what they do in shaping the normative terms through which we pursue LGBT rights in law reform and the critical terms through which we engage in critiques of LGBT rights.

By weaving together critical race, feminist, queer affect and pedagogy scholarship through my autoethnographic account of teaching, I was able to foreground how LGBT rights teaching makes space for emotions, how mapping these emotions expose normative, critical, and theoretical tensions, and how these emotions challenge or reify institutionalised power dynamics within and beyond the classroom (Sedgwick 2003; Ahmed 2006; Browne and Nash 2010; Nichols 2018; Brim 2020). Diarising my affective states in teaching Gender, Sexuality and Law highlighted how the array of the emotions I experienced (anger, hope, anxiety, etc.) connected to an array of registers that I occupied (personal, academic, activist, etc.). To think of this as an emotional event – to synthesise from Williams’ and Ahmed’s work discussed above – is to recognise an affective circularity in relation to the bodies of critical race, feminist, queer scholarship I draw on and the bodies of scholars/students encountering LGBT rights. LGBT rights topics generate emotions (failure to prohibit ‘conversion therapy’ generated hurt and anger). Emotions orient and anchor us to a topic (my anger towards the failure to ban ‘conversion therapy’ rendered it important and salient for inclusion in the GSL unit). Emotions give shape to LGBT rights as a pursuit in normative terms (navigating anger at the failure to ban ‘conversion therapy’ led me to discussions with students about why we might turn to law to eradicate these practices). Emotions also shape the critical terms through which we approach LGBT rights law reform (pain and shame emerged when confronting the limits of law at eradicating the pain of internalised homophobia and transphobia that give rise to conversion practices). Emotional grammar captures the descriptive, normative, and critical dimensions of LGBT rights in legal classrooms and this allows us to account for how we might teach it (pain, shame, and anger related to ‘conversion therapy’ formed the basis through which I introduced my class to the abusive dimension of the practice and how we might regulate it and critique attempts at regulation). The interplay of emotions and issues relating to LGBT people functions to affectively cohere what we (as GSL teachers) conceive of when we speak of LGBT rights and contour the possibilities of how we pursue and critique those rights.

Mapping the emotional grammar of LGBT rights in law schools is important if we seek to grasp the relationship between the pedagogic, political, scholarly, and personal registers of our work and how our emotional investments in each of those registers animate LGBT rights knowledge. Using autoethnography and critical reflections on existing GSL and Law and Emotion scholarship, I have shown the reasons LGBT rights has emerged as an object of study in English law schools and indexed these to an emotional grammar that highlights those disparate reasons. Scholarly research agendas derived from critical race, feminist, and queer research are shaped by feelings of anger, fear, anxiety, desire, and hope to expose, critique, and remedy colonial, patriarchal, and heteronormative legal arrangements. Academics who are LGBT might, as I did, also have emotional investments in making space to represent LGBT people (and their identities, intimacies, injuries), since they have experienced their own pain by being marginalised in/with/through law. We might also have activist/political hopes to promote awareness of non-normative sexualities and genders in order to build a more just world. As teachers, our pedagogic hopes for engendering critical thinking inform how we facilitate a study of how law impacts on minoritised sexualised and gendered communities. By tracing emotion across each of these registers, and accounting for the grammar of their articulation, we can observe how LGBT rights (as a legal object, legal pursuit, and mode of legal critique) materialise through their affective enunciation within law school (expressed as community connection, pedagogic hermeneutic, doctrinal concept, or activist claim).

Mapping the emotional grammar reveals that LGBT rights refuse to be neatly homed in either law school or in law. For example, promising discussions of love in family law and optimism for law reform to recognise some non-nuclear families risk eclipsing queer intimacies that do not fit within such an emotional frame (such as when we lovingly speak about marriage equality). Our emotional attachments to advancing LGBT rights by working within law and law schools might end up as a “restoration” project for a harmful institution that marginalises LGBT people (Ahmed 2019, 217). This connects to the ambivalence evident in my accounts of teaching which reveal LGBT scholars and LGBT rights never neatly fit in law school. Our attachments to the wellbeing of LGBT people and challenging biases in law mean we can create space in law for discussing issues that harm minoritised communities and think about reforms that can ameliorate those harms. For example, emotions like love offer us the opportunity to open up spaces to celebration of law reform (like marriage equality) that affirm the dignity of same-sex relationships. An anxiety about the narrow framing of love also pushed me within the classroom to critique and challenge the capacity of law reforms to redress stigmas directed at gay people and homophobia more generally. I invited students to reflect on whether their optimistic attachments to law reform sustain a fantasy of legal repair that was impossible (Berlant 2011, 25). Yet, I held anxieties as to how critiques of law reform might exoticise or romanticise queer kinship arrangements as a way to alleviate scholarly anxieties about law’s limits. Mapping the emotional grammar reveals the co-constitution, and tensions between, normative and critical dimensions of LGBT rights in law.

Gaining fluency in the emotional grammar of teaching LGBT rights not only makes apparent how LGBT rights coheres as a troublesome object of legal study but also how we might navigate demands for reform and critique these troublesome legal objects generate. Engagements with conventional legal texts dealing with a particular LGBT rights claim, like judgments, generate emotions ranging from outrage and disgust (when reading a case that disparaged homosexual sadomasochism) to optimism and pride (when reading a case decriminalising homosexuality). Yet, the articulation of those rights, within those texts themselves, also relies on refracting specific emotions (whether it is the judicial disgust expressed towards gay sadomasochism or judicial romanticism of gay relationships). Each of these emotional dynamics then circuit into the classroom as students share their own emotional responses to LGBT rights topics. Law teachers are tasked with managing those emotions (alongside their own) to unpack such LGBT rights topics in a way that captures their personal, scholarly, pedagogic, and political registers. While LGBT rights teaching varies in scope and content across institutions, teaching is characterised by a series of emotional grammars that co-constitute varied normative projects that circumscribe legal pursuits of LGBT rights alongside critical projects that enhance our understandings of LGBT rights. Paying attention to this emotional grammar is vital if we are to better understand LGBT rights as objects of legal teaching/study while navigating commitments to pursuing and critiquing LGBT rights in law.

## Footnotes

1 I use the term ‘LGBT’ as it is the most widely used in LLB course materials in British law schools. However, it important to note variations in the acronym exist across historical and cultural contexts. As such, some ‘LGBT rights’ units may reference intersex people, asexuality, and queer minorities who resist labels. For a broader discussion on the challenges of labels in activist spaces and the problems of tokenism see Thoreson (2014).

2 I use ‘GSL unit’ and ‘LGBT rights unit’ somewhat interchangeably throughout this paper. While it is entirely possible to teach a GSL unit without any mention of LGBT rights, none of the publicly identified units taught in the UK do so. While the scope and content of what is meant by ‘LGBT rights’ vary considerably, they remain a core part of GSL units (alongside issues that typically pertain to the experiences of cis straight women).

3 While it is beyond the scope of this paper, it is important to recognise that this paper sits alongside important scholarly work on decolonising the legal curriculum. There is extensive decolonising curriculum work happening in the UK and abroad (see Adebisi 2020).

4 Sara Ahmed carefully details how specific disciplinary canons and conventions are built on the erasure of women’s intellectual, political, spiritual, and emotional labour. In response to this, she makes a call for a “politics of feminist citation” that confronts these erasures and deliberately foregrounds the work of feminist (and other minoritised) scholars (Ahmed 2017, 14–17). This paper is a response to that call.

5 Sara Ahmed (2017) captures this affective labour with the idea of “sweaty concepts” (13). Concepts are “sweaty” because they gain form from bodies at work in institutions where they are “not at home” and have to struggle to be heard (Ahmed 2017, 13). These concepts carry an emotional currency in their circulation, which is a point I explore further when developing the concept of emotional grammar.

6 This paper resonates with a number of themes in Matt Brim’s book, *Poor Queer Studies*: *Confronting Elitism in the University*. Brim’s work explores how pedagogy functions as an arena for the production of critical queer theory and praxis, one that is inflected by class politics (Brim 2020). In this paper, I use the pedagogic encounter in the GSL classroom to illuminate the praxis of how we think, frame, critique, and pursue LGBT rights in law.

7 This paper uses observations drawn from teaching GSL at Keele University. The observations I make about emotion, however, also resonate with GSL teaching in other law schools and jurisdictions. This paper sits within a research project titled 'Legally Queer', funded by the Association of Law Teachers, where I surveyed GSL teachers in the UK about their emotional experiences of teaching LGBT rights. This project received ethics approval from Keele University.

8 An examination of all UK university websites reveals at least 20 institutions with publicly available catalogues that list law electives with a focus on sex, gender, sexuality, and social justice (though the names of such units may vary). I would like to thank Flick Adams for her research assistance on this.

9 The AHRC Research Centre for Law, Gender and Sexuality was funded from 2004 to 2009 and hosted by Keele University, University of Kent, and University of Westminster.

10 For more information on the Queer Judgments Project, see https://www.queerjudgments.org/.

11 This paper acknowledges there is a rich and contested literature on emotions differentiating concepts like affect, drives, feeling, emotion, and passions. However, for the purpose of this paper, I use the term emotion quite expansively to capture what others might wish to describe as affects, feelings, or passions.

12 This also suggests the need for a more honest engagement with emotion in law school more generally, rather than just in GSL. I have discussed this elsewhere (see Raj 2021).

13 The concept of emotional labour has been theorised extensively in much feminist scholarship (see Hochschild 1983).

14 In a *Feminist Legal Studies* editorial about open access publishing, “wench tactics” are presented as a way of working that involves stalling, resisting, and adapting within specific institutional conditions without resigning or withdrawal (see Fletcher et al. 2017, 18–20). In this paper, I take this as inspiration to think about how GSL scholars who teach LGBT rights use such tactics when working within the law and law school environment but also how these tactics might allow such scholars to think reflexively about those ways of working as well.

15 This method comes from feminist cultural studies (see McRobbie 1982).

16 I have discussed affective racialised politics of queer desire in more detail in the context of online dating (see Raj 2011).

17 Whiteness is not confined to skin colour. Whiteness is a political and cultural schema that privileges certain bodies, tastes, activities, and texts over others (see Ahmed 2007).

18 *Obergefell v Hodges*.

19 madison moore (2018) explores how elements of this kinship are expressed in archives of “fabulousness” involving drag queens, trans performers, and Ball Houses.

20 The Government Equalities Office has recently focussed on two key reform areas relating to LGBT people: (1) creating a simpler mechanism for people to change their legal sex without medical bureaucracy (commonly referred to as a ‘self-declaration’ model); and (2) creating a regulatory framework that would end so-called ‘conversion therapies’ that aim to change a person’s sexual orientation and/or gender identity.
